# Comparative Effects on Using Bilberry Leaves in Broiler Diet Reared under Thermoneutral Conditions vs. Heat Stress on Performance, Health Status and Gut Microbiota

**DOI:** 10.3390/life14010039

**Published:** 2023-12-25

**Authors:** Mihaela Saracila, Arabela Elena Untea, Iulia Varzaru, Tatiana Dumitra Panaite, Petru Alexandru Vlaicu

**Affiliations:** 1 Food and Feed Quality Department, National Research and Development Institute for Biology and Animal Nutrition, 077015 Balotesti, Romania; arabela.untea@ibna.ro (A.E.U.); iulia.maros@ibna.ro (I.V.); alexandru.vlaicu@outlook.com (P.A.V.); 2 Nutrition Physiology Department, National Research and Development Institute for Biology and Animal Nutrition, 077015 Balotesti, Romania; tatiana.panaite@ibna.ro

**Keywords:** broiler, intestinal microflora, bilberry leaves, heat stress, thermoneutral condition

## Abstract

The study aims to investigate the impact of dietary bilberry leaves on the performance, health status, and gut microbiota of broilers reared in both thermoneutral conditions and under heat stress. Sixty Cobb 500 broiler chicks were divided into two groups (C-TN, BL-TN) and reared in thermoneutral conditions for the first trial. For the second trial, two other groups (C-HS and BL-HS) were reared in heat stress (32 °C), with 30 chickens in each group. The experimental diets were supplemented with 1% bilberry leaves compared to the control diets. The broilers fed a diet with bilberry leaves had lower levels of cholesterol compared to the control birds. At the end of the experiment, six broilers per group were slaughtered, and intestinal contents were collected for bacteriological analyses. The results revealed that bilberry leaves increased body weight and average daily feed intake in the BL-TN group compared to the C-HS group. However, the broilers fed a bilberry leaves diet and reared in heat stress had a significantly lower average daily feed intake and average daily weight gain than the C-TN group. Additionally, the number of staphylococci colonies decreased significantly in the group fed with a BL-supplemented diet and reared in TN compared to C-TN, while lactobacilli increased significantly in BL-TN compared to C-TN. In summary, bilberry leaves can be used as a natural supplement in a broiler’s diet to regulate serum cholesterol in heat stress and maintain the health of intestinal microflora in thermoneutral conditions.

## 1. Introduction

In the current context, rising temperatures due to global warming caused by increasing industrialization and environmental degradation have led to heat stress becoming a major problem in animal husbandry, particularly in the poultry sector. The lack of sweat glands, the rapid growth, the increase in productive performance, and the efficiency of food conversion makes today’s hybrids much more sensitive to heat stress [1]. Among the negative effects of heat stress are the disruption of the balance between antioxidants and reactive oxygen species, impaired metabolic and immune function, reduced performance and increased mortality and, last but not least, changes in the bacterial composition of the intestine [2]. Of all the organs, the gastrointestinal tract is considered one of the most affected during the exposure of chickens to heat stress [3].

Given the negative consequences of heat stress, the issue of combating them has quickly become of particular interest in the field of animal husbandry. The use of bioactive compounds from natural resources in the diets of broilers to mitigate the impact of high temperatures has been an area of significant interest for poultry producers and nutritionists in recent years.

The bilberry, also known as the European blueberry, is a small shrub that grows naturally belonging to the *Vaccinium* genus of the *Ericaceae* family. While the berries are the main product harvested from the bilberry, recent research has shown that the leaves and stems of the plant contain a significantly higher amount of phenolic compounds, which are known for their antioxidant properties [4]. The aerial parts of the plant are undoubtedly a valuable source of bioactive natural products that can be effectively utilized for the development of food supplements, nutraceuticals, or functional foods. In European countries, tea made from bilberry leaves has a history of traditional use in treating diabetes and urinary diseases because of its astringent and antiseptic properties, as noted by [5]. Bilberry leaves are also being studied for their potential benefits in broiler diets due to their high content of polyphenols, zinc, vitamin E, lutein, and zeaxanthin, as well as their antioxidant capacity [6]. These compounds have biological and therapeutic activities such as antioxidant, anti-stress and immunomodulatory properties [7,8]. Some authors [9] reported that the dietary bilberry leaves positively influence the microbiota of laying hens, while others [10] showed that bilberry leaves increased the total polyphenol content, and the zinc, lutein and zeaxanthin concentrations in the egg yolks. However, its effects on heat stress are unclear. To our knowledge, no information is available on comparing the effect of dietary bilberry leaves alone in broilers reared under thermoneutral conditions vs. high heat stress on the intestinal microflora, although the antimicrobial and antioxidant activities of bilberry leaves have been investigated. 

The aim of this study is to evaluate the impact of bilberry leaves (BL) in the diet of broiler chickens on their performance, health status, and intestinal microflora under both thermoneutral conditions (TN) and heat stress (HS).

## 2. Materials and Methods

### 2.1. Experimental Design 

**Figure 1:**
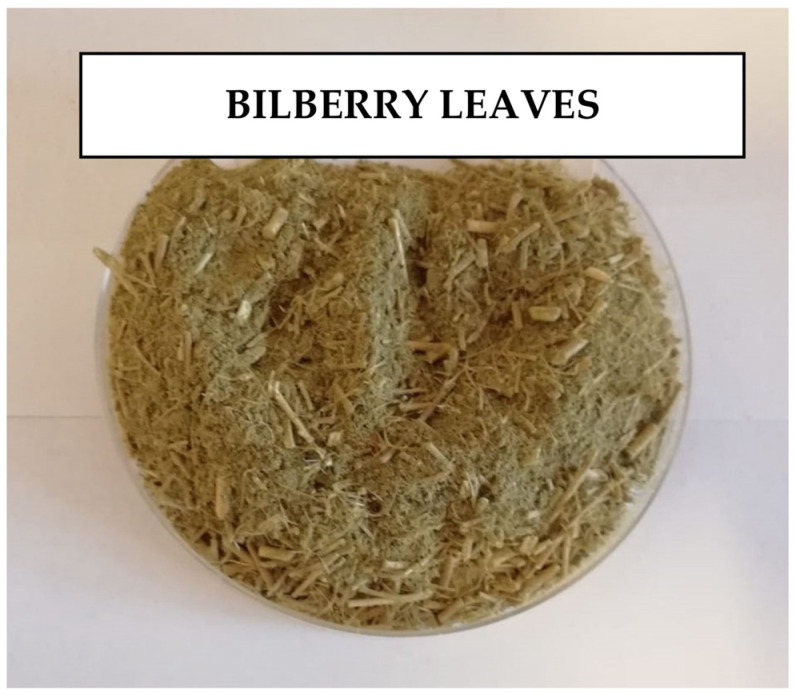
Bilberry leaves.

The feeding trial was conducted in the Laboratory of Chemistry and Nutrition Physiology of the National Research Development Institute for Biology and Animal Nutrition located in IBNA-Balotesti, Romania. The trial was carried out according to a rigorous experimental protocol that was approved by the Ethics Commission of the Institute. A total of 120 Cobb 500 unsexed broilers were involved in the trial and were divided into two feeding trials, each lasting for 42 days. The broilers were housed in digestibility cages that were environmentally controlled. The chicks were weighed and were assigned to four groups consisting of 30 chicks each. In the first trial, two groups (C-TN and BL-TN) were raised in thermoneutral conditions. In the second trial, two other groups (C-HS and BL-HS) were raised in heat stress (HS) conditions. The temperature of 32 ± 1 °C was maintained constant for 42 days. The lighting schedule of 23 h of light and 1 h of darkness was suitable for the broilers’ age. The diet structure was the same for both experiments and met the nutritional requirements specified by the National Research Council (NRC, 1994) and the Cobb 500 hybrid (as shown in Table 1). The control groups (C-TN, C-HS) were given a conventional feed consisting of corn and soybean meal. On the other hand, the experimental diets (BL-TN, BL-HS) had 1% bilberry leaves (as shown in Table 1) added to them. The bilberry leaves (Figure 1) were purchased dried, ground, and packed from pharmacies. Feed and water were provided for them to consume ad libitum.

### 2.2. Performance Parameters

During the experimental period, which lasted from day 1 to day 42 of the broiler’s life cycle, we monitored the following variables: body weight (BW) in grams, average daily feed intake (ADFI) in grams of feed per broiler per day, average daily weight gain (ADWG) in grams per broiler per day, and feed conversion ratio (FCR) in grams of feed per gram of gain. We recorded the individual body weight of each broiler on a weekly basis.

Average daily polyphenols intake (ADPI) for each group was calculated based on the total polyphenol content of feed compounds (mg GAE/g feed compound) and the average daily feed intake of broilers (g/day) using the following formula:

$$ADPI\;\left(mg\frac{TPC}{day}\right)=\frac{Total\;polyphenols\;content\;of\;feed\;compund\;\left(mg\;GAE\right)\times Average\;daily\;feed\;intake\;of\;broiler\;\left(\frac{g}{day}\right)}{Weight\;of\;feed\;compound\left(g\right)}$$

### 2.3. Chemical Analysis of Bilberry Leaves 

#### 2.3.1. Proximate Analysis of Bilberry Leaves and Feed Samples

Measurements and recordings of dry matter, crude protein, ether extractives, crude fiber, and ash levels were conducted and documented in accordance with ISO standards.

#### 2.3.2. Preparation of Methanolic Extract of BL

To obtain extracts, 1 g of sample was added to 10 milliliters of 80% methanol. The samples were then placed on a rotary shaker in a dark environment for 24 h. Afterward, the samples were centrifuged (1500× *g*, 10 min) and the resulting supernatant was used for further analysis.

#### 2.3.3. Quantification of Total Phenolics

The Folin–Ciocalteu colorimetric method was used via spectrophotometry [10] to determine the total phenolic concentration of the bilberry leaves and feed samples. The absorbance was recorded at 732 nm and a standard solution of gallic acid was utilized. Total phenolic concentration is expressed as mg GAE/g using a gallic acid standard curve.

#### 2.3.4. Determination of Total Flavonoids 

Total flavonoid content was determined by the aluminum chloride colorimetric method described previously [11] with slight modifications. The UV-VIS spectrophotometer (JASCO V-560, Japan Servo Co. Ltd., Tokyo, Japan) was used to record the absorbance of the samples against the blank at 410 nm. Quercetin was used as the standard to plot the calibration curve. The flavonoid concentration was expressed as mg Quercetin equivalents (QE) per g.

#### 2.3.5. Lutein and Zeaxanthin

The lutein and zeaxanthin concentrations were determined using a high-performance liquid chromatograph (HPLC) series 200 (Perkin Elmer, Shelton, CT, USA), following the method outlined by [6].

#### 2.3.6. Vitamin E

The concentration of vitamin E was quantified by utilizing a high-performance liquid chromatography technique according to [6]. The HPLC system used for this purpose was a Finningan Surveyor Plus from Thermo-Electron Corporation, located in Waltham, MA, USA. The amount of vitamin E was detected using a specialized PDA-UV detector at a specific wavelength of 292 nm. Finally, the quantitative results were reported in terms of milligrams (mg) of vitamin E per kilogram (kg) of the sample.

#### 2.3.7. Mineral Micronutrients

Mineral micronutrients (Cu, Fe, Mn, Zn) were microwave-digested and then quantified using the method described by [12] with a flame atomic absorption spectrometer (FAAS) Thermo Electron—SOLAAR M6 Dual Zeeman Comfort (Cambridge, UK). Results were reported as milligrams (mg) of vitamin E per kilogram (kg) of the sample.

#### 2.3.8. Determination of Antioxidant Activity

##### DPPH Free Radical Scavenging Assay

The inhibition potential of a sample against DPPH radical was assessed using the method described by [10] with slight modification. A calibration curve was created by using 6-hydroxy-2,5,7,8-tetramethylchroman-2-carboxylic acid—commonly known as Trolox—as the standard. This was carried out to measure the ability of the samples to scavenge radicals in comparison to Trolox. The results were presented in millimoles of Trolox equivalents per kilogram of sample, indicating the sample’s antioxidative capacity.

##### ABTS Free Radical Scavenging Assay

The analysis aimed to determine how effective polyphenolic extracts are at scavenging ABTS radicals. To conduct the analysis, a method described by [10] was followed, which involved measuring the ability of antioxidants to scavenge ABTS radicals generated in the aqueous phase. The method measured the reduction in absorbance resulting from the hydrogen-donating antioxidants present in the extract against a blank at 734 nm using a UV-VIS spectrophotometer (JASCO V-560, Japan Servo Co. Ltd., Tokyo, Japan). The results were expressed as the Trolox equivalent antioxidant capacity in mM (mM TEAC) to quantify the ABTS scavenging activity of the extract. This same measure was also used to express the scavenging effect of the extract on ABTS free radicals.

##### Phosphomolibdenum Method

The antioxidant capacity of bilberry leaves and feed samples was measured using the phosphate-molybdenum method according to [13] with slight modification, which is a common method used to determine the antioxidant capacity in various plant materials. In this method, a reducing agent, also known as an antioxidant, is added to the sample, which reduces molybdenum(VI) to molybdenum(V). The reduction process is followed by the formation of a phosphate/molybdenum(V) green complex with an acid pH. The intensity of the green color is directly proportional to the concentration of antioxidants present in the sample. The results were expressed as mmol AA equivalent/kg DW and as mmol vitamin E equivalent/kg DW, which are commonly used units for expressing the antioxidant capacity of samples.

##### Iron (Fe^2+^) Chelating Activity

In order to determine the chelating effect on ferrous ions, a method described in [14,15] with slight modification was utilized. This method relies on measuring the absorbance of the purple color of the complex formed when an extract competes with ferrozine for ferrous ions. This was carried out by using a UV-VIS spectrophotometer (JASCO V-560, Japan Servo Co. Ltd., Tokyo, Japan) to measure the absorbance against a blank at 562 nm. The Iron (Fe^2+^) chelating activity was calculated by expressing it as mg equivalents of disodium ethylenediamine tetra-acetic acid (EDTA-Na_2_) per gram of the sample (mg equiv. EDTA/g). Similarly, the chelating effect on ferrous ions was expressed as mg equivalents of EDTA per gram of the sample (mg equiv. EDTA/g). This provides a clear and accurate measure of the chelating effect of the extract on ferrous ions.

### 2.4. Sample Collection 

Six chickens per group were slaughtered on day 42 by cervical dislocation and immediately bled. After each bird was euthanized, the gut, from the esophagus to the cloaca, was taken out. Samples of the intestinal content were carefully collected and then placed into sterilized stool collection tubes. These tubes were then stored at a temperature of −20 °C to preserve the samples for further analysis. The analysis focuses on determining the number of *Enterobacteriaceae*, *E. coli*, staphylococci, lactobacilli, and the presence of *Salmonella* spp. in the samples.

### 2.5. Serum Biochemical Attributes

In this study, blood samples were collected 42 days after treatment from six birds per group. The samples were collected from the sub-axial region and stored in heparinized test tubes. The samples were then centrifuged and the serum was separated and stored at a temperature of −20 °C for further analysis. The analysis included measurements of various serum metabolites such as glucose, cholesterol, albumin, creatinine, urea, phosphorus, magnesium, iron, total protein, total bilirubin, alanine aminotransferase, aspartate aminotransferase, and gamma GT. These measurements will provide valuable insights into the potential effects of the treatment on the health of the birds.

### 2.6. Intestinal Microflora Analysis

The intestinal content was analyzed for the quantification of various bacterial species including *Enterobacteriaceae*, *E. coli*, staphylococci, lactobacilli, and *Salmonella* spp. The analysis was conducted using the method described previously [16]. A Scan 300 colony counter (Interscience, Paris, France) was used to count the colonies of bacteria. The number of colonies was expressed as log base 10 colony-forming units (CFU) per gram of intestinal content. This analysis helped in determining the bacterial population in the intestinal content and identifying the presence of any harmful bacteria such as *Salmonella* spp.

### 2.7. Statistical Analysis

The production performance data of all birds, along with their biochemical indices and heat stress response, were analyzed using a 2-way ANOVA with Graph-Pad Prism v. 9.02 (San Diego, CA, USA). The analysis considered the effects of two factors: diet (C, BL) and temperature (TN, HS). Whenever significant effects were observed, individual treatment differences were compared using Tukey’s range test. A significance level of *p* < 0.05 was set.

## 3. Results and Discussion

### 3.1. Chemical Composition of Bilberry Leaves

Table 2 shows the chemical composition of the bilberry leaves. Upon analysis of bilberry leaves, it was discovered that the levels of crude protein and crude fiber were remarkably high, reaching 5.91% and 32.67%, respectively. Furthermore, the supplement BL contained significant amounts of polyphenols, lutein, and zeaxanthin, as well as vitamin E.

The mineral concentration of BL was also found to be high in Mn and Fe. These findings are consistent with [17]. The antioxidant potential of BL was deemed significant due to the phenolic compounds contained in bilberry leaves, which are directly linked to their antioxidant power. Any alterations in the phenolic composition could lead to variations in the in vitro antioxidant activity. Some authors [5] have documented that bilberry leaves possess a rich abundance of microelements, such as Mn (240.9 mg/100 g), Al (24.1 mg/100 g), and Fe (9.05 mg/100 g), indicating their tremendous value as a mineral source. According to [5], the Mn content in the leaves was ten times higher than that in the berries. The mineral content of leaves enhances their nutritional value. Some authors [18] unequivocally proved that leaves contain a significantly higher concentration of polyphenols than berries. Furthermore, their findings indicate that the antioxidative activities of leaves are markedly superior to those of berry extracts, which is undoubtedly a result of the higher content of phenolic compounds present in the leaf extracts [18]. In this line, much information regarding the antioxidative potential of BL was found [19,20]. A study conducted by [21] clearly indicates that BL is more effective in preventing lipid oxidation in meat models. 

Table 3 presents the levels of polyphenols and antioxidants in the feed compounds. The outcome shows a noteworthy improvement in both total polyphenol content and antioxidant capacity when BL was incorporated into the diet. 

### 3.2. Serum Metabolites

Serum biochemical parameters can be used to assess the impact of bilberry leaves on broilers’ metabolism, nutrition, immunity, and growth conditions. The status of an animal’s health is directly reflected in these parameters. The serum biochemical parameters, as shown in Table 4, indicate several advantages of feeding bilberry leaves to broilers.

The inclusion of bilberry leaves into the feed compound of chickens exposed to heat stress has numerous benefits for their biochemical parameters, except for glucose, which displays no significant differences between groups (Table 4). However, the addition of bilberry leaves to their diet led to a substantial decrease in cholesterol levels. Despite this, temperature significantly affected the cholesterol level in their blood, as chickens raised in heat stress exhibit higher cholesterol levels compared to those raised in normal temperatures, irrespective of their diet (C or BL). It is a well-established fact that stress can significantly increase total cholesterol levels in the body, leading to a higher risk of cardiovascular diseases. Nonetheless, it is worth noting that, regarding most bird species, the plasma cholesterol concentrations are typically within the range of 100 to 250 mg dL^−1^ [22]. In this study, it was found that, even with varying ages and environments, the cholesterol concentration persisted within the normal range for the species. The use of *Vaccinium myrtillus* leaves as an antidiabetic treatment has been limitedly investigated and the results have been inconsistent. In one study [23], researchers administered a dry hydroalcoholic extract of *V. myrtillus* leaves to diabetic streptozotocin rats, resulting in a significant reduction of blood triglycerides, by 39%. A research study [24] has highlighted that bilberries, due to their rich anthocyanin content, could potentially be an effective dietary preventative measure against hypercholesterolemia in rats. This finding suggests that incorporating bilberries into a balanced diet may have potential health benefits. However, the decrease in plasma glucose levels by 26% was deemed statistically insignificant. Another study by [25] tested a multi-ingredient preparation containing *Myrtilli folium* and nine other plant extracts on alloxan-induced non-obese diabetic mice. The results showed a reduction in both blood glucose and fructosamine levels.

There was no noticeable difference in renal, liver, and mineral metabolism with the addition of Bl supplementation. Furthermore, albumin levels were significantly affected by temperature, with higher levels observed in chickens from group C raised under heat stress in comparison to chickens from C (TN) and BL (TN). Correspondingly, creatinine levels were significantly higher in chickens raised in heat stress (C and BL) than in chickens from C raised in normal temperatures. The urea level was influenced by both diet and temperature, with significantly higher levels observed in BL group chickens raised in heat stress compared to those raised in normal temperatures (C and BL). The findings are consistent with those presented in [26].

As for the mineral profile, only temperature had a significant influence on the levels of phosphorus and magnesium. Phosphorus levels were significantly higher in chickens raised in heat stress (groups C and BL) than in group C, raised in normal temperatures. Magnesium levels were detected in significantly lower concentrations in chickens raised in heat stress than those raised in normal temperatures, while the level of Fe was influenced by both diet and temperature, with significantly higher levels observed in the BL group chickens, raised in heat stress, compared to those raised in normal temperatures (C and BL). There were no significant differences in the levels of total protein between the groups at any temperature with respect to liver parameters. Total bilirubin levels were significantly higher in group C (HS) than in group BL (TN). In this study, ALT, AST, and gamma GT range values were significantly higher in C and BL groups under heat stress compared to C and BL at normal temperatures. Elevated levels of ALT and AST in serum are clear indications of tissue damage, specifically in the liver and muscle [27]. When an organism is exposed to high temperatures, a phenomenon known as heat stress occurs. As a result of this stress, the levels of free radicals in the body increase significantly. At the same time, the activities of antioxidant enzymes that usually help in scavenging these harmful molecules decrease, leading to an accumulation of free radicals. This has been demonstrated by [28], who found that, during heat stress, the body’s free radical scavenging ability is compromised. When broiler chickens are exposed to high environmental temperatures, it can cause damage to their liver tissues through oxidative stress. This can lead to further complications with their lipid metabolism, according to research by [29].

### 3.3. Performance 

**Figure 2:**
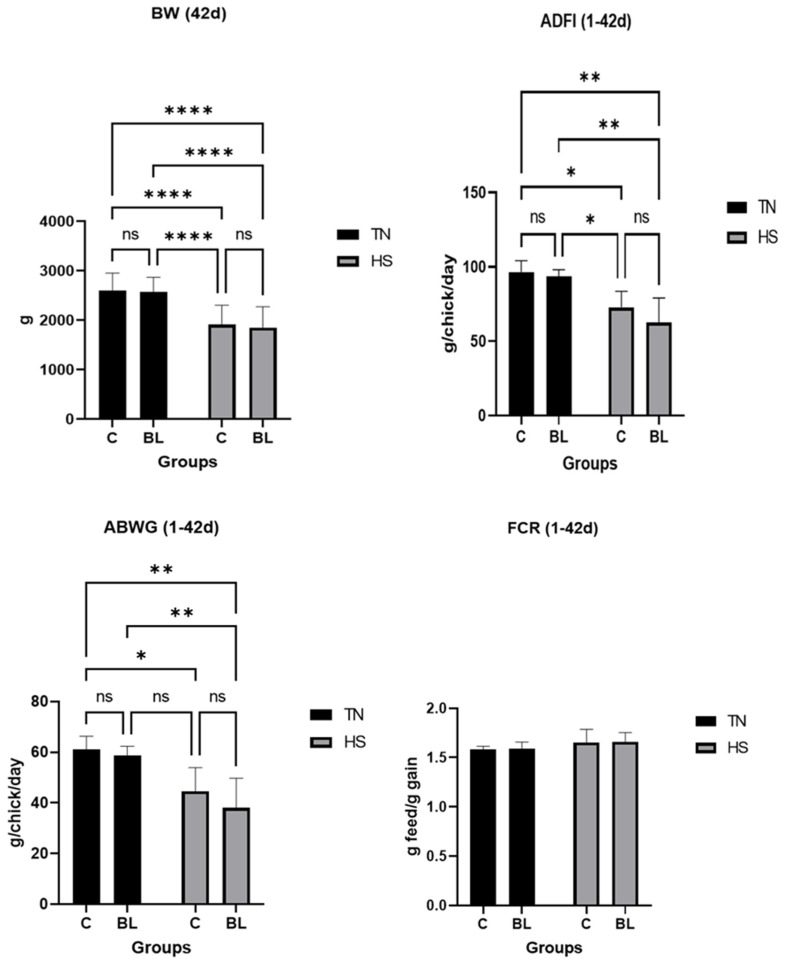
Effects of BL and temperature on productive performance of broilers. *p* > 0.1234 ns-non significant, * *p* ≤ 0.0332, ** *p* ≤ 0.0021, **** *p* < 0.0001; C: control diet; BL: treatment with 1% bilberry leaves.

Figure 2 clearly illustrates the undeniable impact of various diets on broiler performance. Notably, it is evident that the chickens raised in heat stress exhibited a significant decline in their overall performance (in terms of BW, ADFI, and ABWG) compared to those reared under thermoneutral conditions, despite receiving identical ratios. This outcome was entirely predictable and serves as a reminder of the importance of maintaining optimal conditions for broiler growth and development. Groups C and BL exhibited a substantial reduction in BW in HS in comparison to TN. The effect of the BL diet on BW was not significant in both HS and TN. However, the BL (TN) group saw an increase in BW relative to the C (HS) group. The average daily feed intake (ADFI) was found to be greater in the BL (TN) group as compared to the C (HS) group. This indicates that the BL (TN) group consumed a larger quantity of feed on a daily basis than the C (HS) group. The broilers that received BL as a supplement and were raised in HS (high stress) conditions exhibited a marked decrease in both their average daily feed intake (ADFI) and average daily body weight gain (ABWG) when compared to those from the control group (C) that was raised in TN (normal conditions). The results of the 42-day experiment indicate that there were no significant differences in the feed conversion ratio (FCR) between the two conditions of thermal neutral (TN) and heat stress (HS) (*p* > 0.05). There have been only a few studies investigating the impact of dietary bilberry leaves on broiler productive parameters. Some authors [30] discovered that a dietary phytogenic mixture containing bilberry leaves did not affect the performance of broilers, regardless of temperature conditions. The same trend was observed by [17], which demonstrated that dietary bilberry leaves (0.5%) did not significantly contribute to the productive parameters of laying hens under normal temperatures.

**Figure 3:**
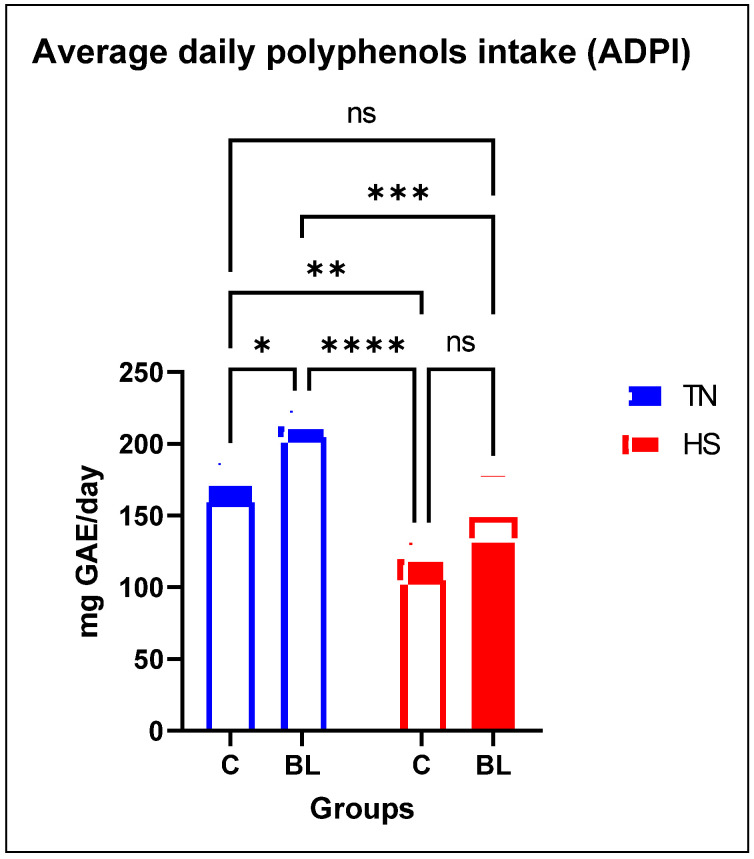
Average daily polyphenol intake (mg GAE/day) of broiler chickens. *p* > 0.1234 ns-non significant, * *p* ≤ 0.0332, ** *p* ≤ 0.0021, *** *p* ≤ 0.0002, **** *p* < 0.0001; C: control diet; BL: conventional diet + 1% bilberry leaves.

Figure 3 showed the average daily polyphenol intake of broilers. Both diet and temperature had a significant effect on ADPI. The results showed that broilers from the BL group reared under TN had a higher ADPI than those from the other groups. This was due to the effect of the temperature, which decreased feed consumption, and therefore also polyphenol intake. 

### 3.4. Intestinal Bacterial Populations

Table 5 demonstrates that the addition of dietary supplements did not have any significant effect on the number of *Enterobacteriaceae* and *E. coli*. However, higher temperatures were observed to have a significant effect on the growth of these bacteria, resulting in increased bacterial numbers.

The presence of staphylococci and lactobacilli was influenced by both diet and temperature (Table 5). In TN, the addition of BL in the diet has a positive effect, leading to a significant decrease in the number of staphylococci colonies compared to the control group (C). Conversely, high temperatures resulted in an increase in the number of staphylococci and a decrease in the number of lactobacilli in the intestine (Table 5). Nonetheless, lactobacilli increased in the group that included BL in the diet (TN) compared to the control group (C), which means that BL promoted the multiplication of these probiotic bacteria.

**Figure 4:**
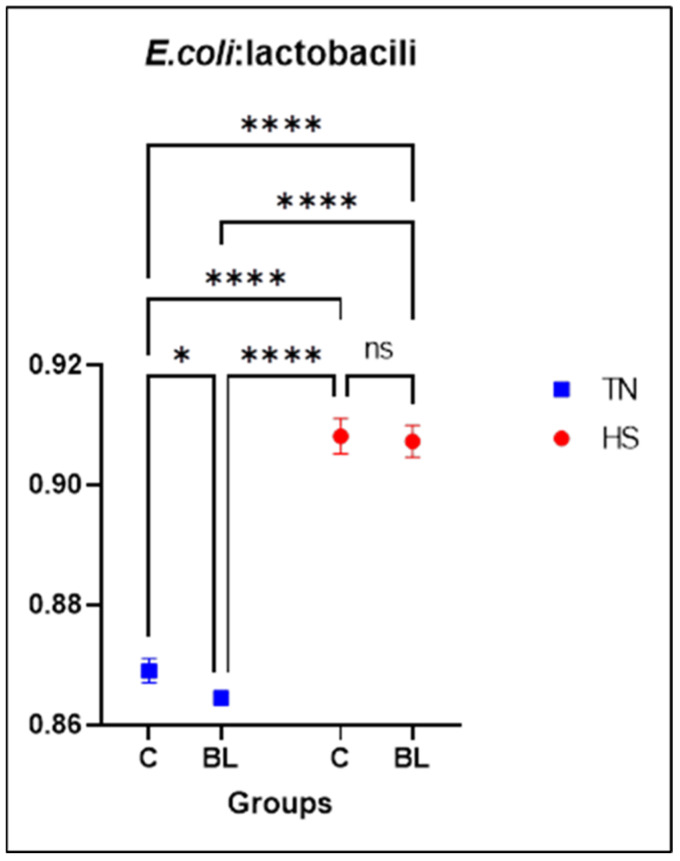
The effect of BL and temperature on the *E. coli*: lactobacilli ratio in the intestinal content of chickens. *p* > 0.1234 ns-non significant, * *p* ≤ 0.0332, **** *p* < 0.0001; C: control diet; BL: conventional diet + 1% bilberry leaves.

In Figure 4, the effect of the tested diet and temperature on the *E.coli*: lactobacilli ratio in the intestinal content at 42 days is shown. It is observed that supplementing the diets with BL did not significantly affect the ratio of *E. coli*: lactobacilli in HS. But in TN, the addition of BL significantly decreased the ratio of *E. coli*: lactobacilli, thus the addition of BL positively affected it. These results could be due to the antioxidant effect of the bioactive compounds contained in BL, which inhibit the growth of bacteria and have a probiotic-like effect. Several studies have shown that the phenolic compounds present in bilberry leaves can reduce pathogenic bacteria in vitro, suppressing both gram-negative and gram-positive bacteria [4]. Beneficial bacteria play an important role in various biological functions in the body. They produce digestive enzymes, boost leukocyte activity, and provide essential vitamins and amino acids [31]. Changes in the gut microbiota composition are also linked to oxidative stress [32].

Herbs and natural products can achieve antimicrobial effects through the inhibition of bacterial adhesion to cell walls, direct antimicrobial killing, or by enhancing the effects of antibiotics. One study demonstrated that dietary supplementation with bilberry extract significantly decreased the size of *Enterobacteriaceae* populations in broilers. Some authors showed that diets enriched with bilberry and walnut leaves powder positively altered the microbiota of hens by modulating several digestive enzymes, leading to increased lactobacilli and decreased *Enterobacteriaceae* [9].

There are various ways to inhibit the growth of harmful bacteria, each with a unique approach. One of the methods is destabilizing the cytoplasmic membrane, which involves breaking down the membrane barrier surrounding the bacterial cell. Another method is permeabilizing the plasma membrane, which weakens the membrane and makes it easier for antimicrobial agents to penetrate the cell. Additionally, hindering extracellular microbial enzymes can be an effective way to prevent bacterial growth. Another approach is to directly affect microbial metabolism, which can stop the bacteria from producing energy or synthesizing essential proteins. Lastly, depriving the necessary substrates for microbial growth can also halt bacterial growth. These methods are crucial for preventing the spread of bacteria and ensuring a safe environment for all [33]. Several studies have confirmed the efficacy of leaves and pomaces in fighting pathogens (refer to Table 5) [34,35,36]. Notably, bilberry leaf extract has been found to be more effective than fruit extracts in inhibiting the growth of both methicillin-resistant and methicillin-sensitive *S. aureus*, according to [37]. This is likely due to the significant amount of chlorogenic acid present in both the leaf and berry extracts. Meanwhile, some researchers [18] discovered that Finnish berry species have varying degrees of resistance to different strains of bacteria, including *Bacillus cereus*, *Listeria monocytogenes*, *S. aureus*, *E. coli*, and *S. enterica sv*. *Typhimurium*. While most cases showed clear inhibitory effects when 20 lL of extract was added to a 300 lL culture medium, bilberry and chokeberry leaf extracts failed to inhibit *E. coli* growth at low dosages (10 lL), and only moderate inhibition was observed with berry extracts [38]. Furthermore, the study found that *B. cereus* strains were not sensitive to any extracts from these two species at a dosage of 10 lL. Identifying the primary components responsible for the anti-bacterial effects of berry extracts can be challenging, especially when considering that a low pH value also contributes to the inhibition of bacterial growth. It has been conclusively established by researchers that bilberry leaves possess potent antistaphylococcal properties, which effectively inhibit the growth of *S. aureus* [37]. Furthermore, there is irrefutable evidence to suggest that, when used in combination with vancomycin and linezolid, bilberry leaves significantly augment the bactericidal potential of these antibiotics [39]. 

## 4. Conclusions

In summary, the current findings suggest that dietary bilberry leaves can contribute to the health of chickens challenged by heat stress through its cholesterol-lowering effect. This has major relevance in the poultry sector, especially in the context of global warming. In addition, dietary bilberry leaves can potentially be used as a natural and effective dietary supplement in the broilers’ diet, with a positive effect on intestinal microflora in thermoneutral conditions without affecting performance. However, there is a need for comprehensive studies validating its dose and mode of action in broilers exposed to heat stress.

## Figures and Tables

**Table 1:** Diet formulation.

Ingredient	Grower (14–28 d)		Finisher (28–42 d)	
	C	BL	C	BL
Corn	36.47	35.47	40.45	39.45
Wheat	20.00	20.00	20.00	20.00
Corn gluten, 65% CP	4.00	4.00	6.00	6.00
Soybean meal, 46% CP	30.2	30.2	23.95	23.95
Sunflower oil	4.31	4.31	4.72	4.72
*Bilberry leaves*	-	1.00	-	1.00
Monocalcium phosphate	1.52	1.52	1.43	1.43
Calcium carbonate	1.38	1.38	1.31	1.31
Salt	0.38	0.38	0.33	0.33
Methionine	0.25	0.25	0.21	0.21
Lysine	0.29	0.29	0.36	0.36
Choline	0.05	0.05	0.05	0.05
Vitamin-mineral premix	1.00	1.00	1.00	1.00
TOTAL	100	100	100	100
Calculated				
Metabolizable energy, kcal/kg	3128.99		3217.72	
Crude protein, %	21.50		20.00	
Ether extractives, %	6.01		6.49	
Crude fibre, %	3.57		3.36	
Calcium, %	0.87		0.81	
Phosphorus, %	0.70		0.65	
Available phosphorus, %	0.43		0.41	
Lysine, %	1.29		0.16	
Methionine, %	0.61		0.32	
Tryptophan, %	0.22		1.19	

**Table 2:** Data on the chemical composition of bilberry leaves included in the diets.

Parameter	Bilberry Leaves (BL)
Proximate composition	
DM, %	91.67
CP, %	5.91
EE, %	1.57
CF, %	32.67
Ash, %	3.05
Bioactive compounds	
TPC, mg GAE/g	35.90
TFC, mg QE/g	5.54
Vitamin E, ppm	111.28
Lutein + zeaxanthin, ppm	70.59
Minerals	
Cu, ppm	6.27
Fe, ppm	83.09
Mn, ppm	2189.50
Zn, ppm	34.27
Antioxidant activity	
DPPH, mM Trolox	647.88
ABTS, mM Trolox	1.39
TAC, mM ascorbic acid equivalent	301.46
TAC, mM vitamin E equivalent	184.15
Fe chelating power, mg/g equiv. EDTA	1.39

DM—dry matter; CP—crude protein; EE—Ether extractives; CF—crude fiber; TPC—total polyphenol content; TFC—total flavonoids content; TAC—total antioxidant capacity; GAE = gallic acid equivalents; QE = quercetin equivalents.

**Table 3:** Total polyphenol content and antioxidant capacity of feed compounds.

Specification	C	BL	SEM	*p*-Value
Total polyphenols				
TPC, mg/g GAE	1.79 ^b^	2.27 ^a^	0.143	0.0286
Antioxidant capacity				
TAC, mM ascorbic acid equivalent	29.60 ^b^	31.79 ^a^	0.759	0.0335
TAC, mM vitamin E equivalent	30.12 ^b^	33.20 ^a^	0.820	0.0356

TPC—total polyphenol content; TAC—total antioxidant capacity; ^a,b^ Means within a row with no common superscript differ (*p* < 0.05); and SEM = standard error of the mean.

**Table 4:** Effects of BL and temperature on serum metabolites of broilers in TN and HS.

	TN		HS		*p*-Values Summary		
	C	BL	C	BL	Diet	Temp.	Diet × Temp.
Energy parameters							
Glucose mg/dL	212.9	222.6	246.4	237.5	ns	ns	ns
Cholesterol mg/dL	150.7 ^a^	144.6 ^c^	171.6 ^b^	120.0 ^c^	****	*	***
Renal metabolism							
Albumin, g/L	1.00 ^b^	1.00 ^b^	1.41 ^a^	1.22 ^ab^	ns	***	ns
Creatinine, mg/dL	0.10 ^b^	0.13 ^ab^	0.17 ^a^	0.16 ^a^	ns	***	ns
Urea, mg/dL	2.92 ^b^	3.11 ^b^	4.39 ^ab^	6.36 ^a^	*	***	ns
Mineral metabolism							
Phosphorus, mg/dL	4.89 ^b^	5.34 ^ab^	6.19 ^a^	6.23 ^a^	ns	**	ns
Magnesium, mg/dL	2.45 ^a^	2.50 ^a^	1.36 ^b^	1.28 ^b^	ns	****	ns
Iron, µg/dL	90.09 ^b^	100.2 ^b^	108.2 ^ab^	133.7 ^a^	*	***	ns
Liver parameters							
Total protein, g/dL	2.47	2.69	2.49	2.77	ns	ns	ns
Total bilirubin, mg/dL	0.14 ^ab^	0.11 ^b^	0.61 ^a^	0.49 ^ab^	ns	**	ns
ALT, U/L	7.55 ^b^	9.73 ^b^	38.61 ^a^	32.24 ^a^	ns	****	ns
AST, U/L	70.86 ^b^	69.34 ^b^	494.4 ^a^	418.9 ^a^	ns	****	ns
Gamma-glutamyltransferase, U/L	14.87 ^b^	22.96 ^b^	31.68 ^a^	35.88 ^a^	*	****	ns

AST—aspartate aminotransferase; ALT—alanine aminotransferase; ^a,b,c^ Means within a row with no common superscript differ (*p* < 0.05); and SEM = standard error of the mean; *p* > 0.1234 ns-non significant, * *p* ≤ 0.0332, ** *p* ≤ 0.0021, *** *p* ≤ 0.0002, **** *p* < 0.0001); C: control diet; BL—conventional diet + 1% bilberry leaves.

**Table 5:** Effects of BL and temperature on intestinal bacterial populations (lg10 CFU/g wet intestinal content).

Variable	TN		HS		*p*-Values Summary		
	C	BL	C	BL	Diet	Temp.	Diet × Temp.
*Enterobacteriaceae*	6.60 ^b^	6.64 ^b^	7.41 ^a^	7.41 ^a^	ns	****	ns
*E. coli*	5.64 ^a^	5.61 ^a^	6.21 ^b^	6.20 ^b^	ns	****	ns
Staphyilococi	5.71 ^b^	5.60 ^c^	5.97 ^a^	5.97 ^a^	****	****	****
Lactobacili	7.15 ^b^	7.17 ^c^	6.18 ^a^	6.19 ^a^	****	****	ns
*Salmonella* spp.	absent	absent	absent	absent	NA	NA	NA

^a,b,c^ Means in the same column with different superscripts differ significantly (*p* < 0.05). NA = non-adequate; *n* = 6; *p* > 0.1234 ns-non significant, **** *p* < 0.0001; C: control diet; BL—conventional diet + 1% bilberry leaves; CFU—colony forming units.

## Data Availability

All data are contained within the article.
